# Hand hygiene adherence in intensive care units: comparison before and during the COVID-19 pandemic in a municipality of São Paulo state

**DOI:** 10.31744/einstein_journal/2025AO0951

**Published:** 2025-01-24

**Authors:** Vanessa Aparecida Vilas-Boas, Pedro Antonio Teodoro de Moraes, Marcela Grispino Vieira Torres de Lolo, Edinêis de Brito Guirardello, Maria Isabel Pedreira de Freitas

**Affiliations:** 1 Universidade Estadual de Campinas Faculdade de Enfermagem Campinas SP Brazil Faculdade de Enfermagem, Universidade Estadual de Campinas, Campinas, SP, Brazil.; 2 Prefeitura Municipal de Campinas Secretaria Municipal de Saúde Campinas SP Brazil Secretaria Municipal de Saúde, Prefeitura Municipal de Campinas, Campinas, SP, Brazil.

**Keywords:** Hand hygiene, Hand sanitizers, Risk reduction behavior, COVID-19, Coronavirus infections, Pandemics, Intensive care units

## Abstract

**Objective:**

Compare alcohol-based hand sanitizer use in intensive care units in a municipality in São Paulo state, before and during the coronavirus pandemic.

**Methods:**

Analytical retrospective study using indirect documentation, with data obtained from a notification spreadsheet for epidemiological indicators of healthcare-associated infection in the state. Data on monthly alcohol-based sanitizer use were collected from the intensive care units of public and private general hospitals of the municipality. Analysis was conducted using descriptive statistics and graphically with run charts. The Mann-Whitney test was applied to compare the median consumption of public and private hospitals, at a 5% significance level.

**Results:**

Adult, neonatal and pediatric intensive care units showed median increases in hand sanitizer use of 34.03 to 57.64, 31.53 to 48.66 and 34.38 to 60.35mL/patient-day, respectively. Private hospitals showed greater hand sanitizer use in the adult and pediatric intensive care units compared to public institutions.

**Conclusion:**

The pandemic contributed to increasing hand sanitizer use in municipal intensive care units, but there is still room for improvement. More effort is needed to ensure that hand sanitizer use remains high.

## In Brief

This is a pioneering study on the assessment of a Brazilian municipality entire, comparing alcohol-based hand sanitizer consumption before and during the COVID-19 pandemic. Vilas-Boas et al reported a rise from 24.2mL/PD in 2018 to 46.6 in 2020, being for adult intensive care units the municipality exceeded most of the Brazilian states. However, observed a decline in some hospitals in 2021.

## Highlights

■ The COVID-19 pandemic has increased adherence to hand hygiene in intensive care units.
■ Private hospitals recorded higher consumption of alcoholbased hand sanitizer.
■ Interventions are needed to help keep rates up.

## Graphical abstract

**Figure:**
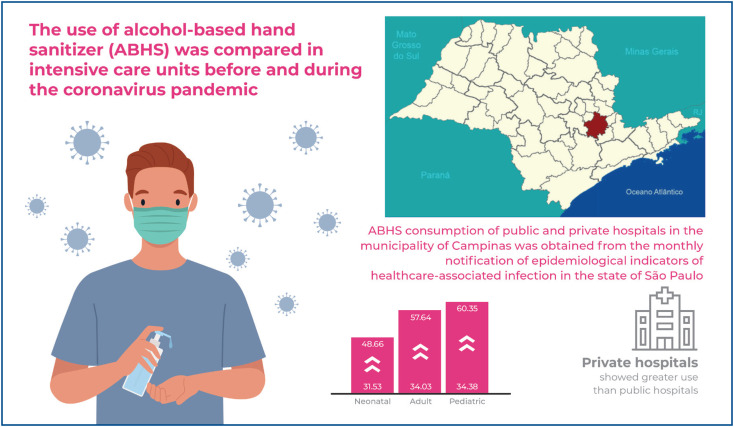

## INTRODUCTION

Hand hygiene is a simple and effective measure to control and combat healthcare-associated infections (HAIs).^(1)^ Correct hand hygiene mitigates microorganism cross-contamination, protecting both patients and health professionals.^(2-4)^ Included as part of Sustainable Development Goal no. 6 - Clean Water and Sanitation, the promotion of hand hygiene is important for achieving other goals, especially Goal no. 3 - Good Health and Well-Being, by helping combat transmissible diseases.^(3-4)^

In this respect, scarce resources, proper sanitation and access to water remain a challenge for implementing an infection prevention and control (IPC) program in many health units located in low-income countries.^(3,5)^ In middle-income countries, even when IPC programs are in place, they often have difficulty functioning properly. According to a World Health Organization (WHO) report, in 2019 there were IPC programs in almost all secondary and tertiary health units, but they had no full-time IPC professionals, allocated IPC budget, routine microbiological laboratory support, or adequate workload, personnel and bed occupancy, exhibiting cost and market limitations.^(3)^

Among the core components of this program are infection prevention and control, education and training, and hand hygiene.^(6,7)^ Infection prevention and control covers clinical and public health practices aimed at reducing the risk of infection and combating the spread of antimicrobial resistance.^(3)^ A study carried out in the eastern Mediterranean to map the education and training components in infection prevention and control found that in 92.9% of cases the training of health professionals depends on the facilities, not on a national education program.^(8)^

In health care, especially in the hospital environment, the WHO recommends that hand hygiene be carried out five moments during patient care: before touching a patient; before a clean/aseptic procedure; after body fluid exposure risk; after touching a patient; and after touching a patient surroundings.^(9)^ Other indications for hand hygiene have also been highlighted, such as at the start and end of a shift, when changing the care site of the same patient, immediately after removing gloves, before preparing food, before preparing and handling medications, before meals and after using the toilet.^(7,10,11)^

However, it is known that this practice is frequently neglected by health professionals^(12,13)^ even in intensive care units (ICUs), with adherence rates varying from 9 to 85%, depending on the geographic region, type of ICU, professional category and the 5 hand hygiene moments.^(13)^ Studies worldwide have also compared the hand hygiene adherence rate during the COVID-19 pandemic with previous years, showing a rise of 16.7 to 45.5% in hospitals.^(14,15)^

COVID-19 is a respiratory disease caused by coronavirus 2, responsible for the severe acute respiratory syndrome (SARS). It originated in December 2019 in Wuhan, China, and was declared a pandemic in March 2020. During the pandemic, hand hygiene was essential in preventing infection and globally acknowledged by policy makers, health administrators, health professionals and the public as a whole.^(1,16)^ An Italian study found alcohol-based hand sanitizer (ABHS) consumption in 27 hospitals, with an increase from 36.1 liters (L)/1000 patient-days (PD) in 2017 to 90.7 in 2020 in ICUs.^(2)^

There are different ways to monitor hand hygiene adherence. Direct observation uses a person to determine whether the health professional correctly executes hand hygiene according to the opportunities and guidelines related to the 5 moments. If observation is open, an immediate correction can be made, thereby guaranteeing adherence. However, both open and secret observation are subject to the Hawthorne effect and selection bias.^(10,17)^ Other forms of observation can be performed by patients or video cameras.^(10)^

Monitoring ABHS use is an indirect method to avoid selection, observer and observation bias and provides hand hygiene estimates and trends, incorporating daytime, nighttime and weekend shifts, without specialized workers. However, it is impossible to determine the performance of the technique, distinguish between professional categories or patients/ visitors, or if hand hygiene is being performed at opportune times, in line with the 5 moments.^(10,17)^ Electronic devices have also been studied and implemented to offset some of the aforementioned limitations, but there is a need for technological and maintenance support and guarantee of privacy, which are considered high-cost.^(10,18-20)^

This study was prompted by the global WHO campaign Save Lives: Clean Your Hands of 2021, which called on health professionals during the COVID-19 pandemic to correctly clean their hands when treating patients.^(1)^

In this respect, liquid, gel or foam ABHS is a determining factor for health professional adherence to the 5 hand hygiene moments since it is easy to provide the product as close as possible to the patient's bed for immediate use. In addition, this product has been highly recommended in most clinical situations, since it is better than hand washing in preserving the skin integrity of health professionals. It also reduces hand sanitizing time, is healthier for the planet and effective in decreasing multidrug-resistant organisms, provided it is used in suitable concentrations to promote microbial death (∼70%) and sufficient amounts to cover the entire hand surface.^(10,17,21,22)^

## OBJECTIVE

To compare alcohol-based hand sanitizer use in intensive care units in a municipality of São Paulo state, before and during the coronavirus pandemic.

## METHODS

### Study design

This is an analytical, retrospective study conducted using indirect documentation and presented according to Strengthening the Reporting of Observational Studies in Epidemiology (STROBE) guidelines.^(23)^

### Study site

The study was conducted in Campinas, São Paulo state, Brazil. According to Brazilian Institute of Geography and Statistics (IBGE - *Instituto Brasileiro de Geografia e Estatística*) data from 2022, Campinas covers 794,571km^2^, with a population of approximately 1,138,309 inhabitants, distributed over four districts. It has a complex health service network that incorporates the National Health System (SUS - *Sistema Único de Saúde*) and the private sector, serving the municipality and region, and is recognized as a regional referral center for 19 municipalities.^(24)^ In 2022, there were 25 hospitals (including 4 public) registered in Campinas and overseen by the Municipal Health Surveillance Department (MHSD).

### Population

The target population consists of general or specialized, public, private or philanthropic hospitals, belonging to the municipality of Campinas, SP, Brazil. Included were hospitals with an ICU that reported ABHS consumption to the MHSD. The following exclusion criteria were adopted: psychiatric hospitals, long-term care institutions and hospitals that provided incomplete data during the study period.

The sample consisted of adult intensive care units (AICU), pediatric intensive care units (PICU) or neonatal intensive care units (NICU). These units were selected for being monitored nationally and internationally by HAI surveillance systems.

### Data collection

Data were collected between November 2021 and April 2022. A four-year period was established, considering the period before (2018-2019) and during the pandemic (2020-2021) for comparison purposes.

Every month, hospitals send the notification spreadsheet for epidemiological indicators of HAI in São Paulo state to the MHSD. This spreadsheet contains the indicators selected by hospital type by the state Epidemiological Surveillance Center for the HAI surveillance system. Briefly, in São Paulo state, hospitals provide the data that is analyzed by the MHSD and then reported to the state, which is responsible for reporting the data to the National Health Surveillance Agency.

The general hospital spreadsheet contains the following indicators: institution characterization; surgical site infections for clean surgeries and other selected surgeries; infections associated with invasive devices in ICUs; ABHS consumption for hand hygiene in ICUs; positive hemocultures, microorganisms and microbial resistance markers for primary bloodstream infection; and antimicrobial consumption. The present study obtained data on ABHS consumption in ICUs.

The spreadsheets are not in the public domain. Authors were given access to a protected folder on Google Drive, after approval from the Municipal Health Surveillance Department and the Research Ethics Committee, respecting the confidentiality of information.

Data extraction, tabulation and primary organization were performed on a Microsoft Excel spreadsheet, which contained the hospital code, type (public or private), number of beds, type of ICU, month and year, volume of ABHS and number of patient-days. Data were collected by a researcher and verified by two others. Cases with missing or inconsistent data were discussed by the research team, following the previously established exclusion criteria.

### Data analysis

In order to calculate monthly ABHS consumption at the unit, total ABHS in millimeters (mL) was divided by the number of patient-days (PD) per month.

Analysis was conducted using the mean, median and interquartile range (IQR), and graphically by run charts. The primary objective of a run chart is to detect process improvement or degradation, which are nonrandom patterns of data point distribution around the median.^(25)^ The median is typically used because it is not influenced by extreme values. Analyses were carried out for each of the hospitals and the municipality, considering the onset of the pandemic as the turning point. The Mann-Whitney test was applied to compare the median consumption values of public and private hospitals, at a 5% significance level. Data were analyzed using SAS 9.4 software.

### Ethical aspects

The study was approved by the Research Ethics Committee of the *Universidade Estadual de Campinas*, CAAE: 51643321.7.0000.5404; # 5.029.924, in line with National Health Council Resolution no. 466/2012.

## RESULTS

**Figure 1:**
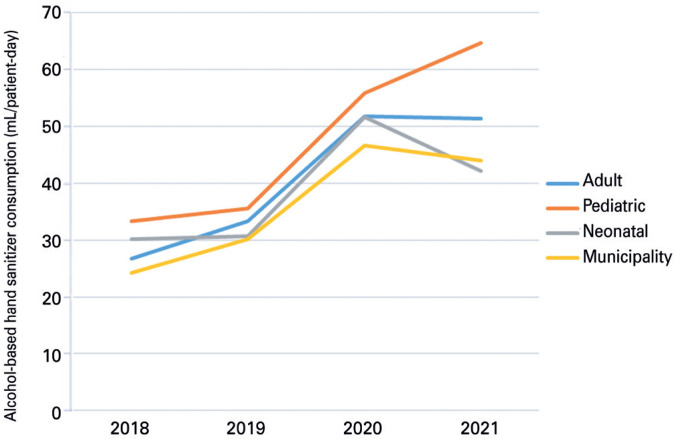
Average annual alcohol-based hand sanitizer consumption, by type of intensive care unit

In 2018, 23 general hospitals reported their data to the municipal Department of Health Surveillance. Two private institutions were excluded for not having an ICU and one that had closed in 2019, one public and one private hospital that provided incomplete data during the pandemic. In addition, one philanthropic hospital was excluded during data analysis to control bias, since it was an outlier. This hospital had only one ICU bed and one PICU bed and always reported its ABHS consumption data as 100mL/PD. Thus, the sample consisted of 17 hospitals, three from the National Health System (SUS) (A1 to A3), and 14 from the private sector (A4 to A17), six of which are affiliated with the SUS (A9, A13 to A17), and five philanthropic institutions (A13 to A17). Table 1 shows the main characteristics of the hospitals and table 2 annual ABHS consumption at the hospitals. Annual average consumption at the ICUs and the municipality are presented in figure 1.

**Table 1:** Characterization of general hospitals in the municipality

Hospital	Type	Affiliated to SUS	Number of beds			
			Total	AICU	PICU	NICU
A1	Public	Yes	142	6	0	15
A2	Public	Yes	219	40	10	0
A3	Public	Yes	219	16	0	10
A4	Private	No	107	19	0	0
A5	Private	No	230	50	8	0
A6	Private	No	24	12	0	0
A7	Private	No	90	18	4	8
A8	Private	No	78	8	2	7
A9	Private	Yes	150	38	8	10
A10	Private	No	44	5	0	3
A11	Private	No	88	20	0	0
A12	Private	No	152	14	6	10
A13	Philanthropic	Yes	77	0	8	0
A14	Private/Philanthropic	Yes	121	30	0	0
A15	Private/Philanthropic	Yes	108	17	0	0
A16	Private/Philanthropic	Yes	212	6	0	36
A17	Private/Philanthropic	Yes	308	36	10	15

Source: The authors, based on data provided by the Municipal Health Department of Campinas, SP, Brazil.

SUS: *Sistema Único de Saúde*; AICU: adult intensive care units; PICU: pediatric intensive care units; NICU: neonatal intensive care units.

**Table 2:** Annual alcohol-based hand sanitizer consumption by hospital and type of intensive care unit

Hospital	Alcohol-based hand sanitizer consumption (mL/patient-day)											
	Adult intensive care unit				Pediatric intensive care unit				Neonatal intensive care unit			
	2018	2019	2020	2021	2018	2019	2020	2021	2018	2019	2020	2021
A1	34.2	27.7	55.6	60.3	(…)*	(…)	(…)	(…)	30.6	42.2	61.2	58.3
A2	7.0	15.2	40.3	22.4	8.5	20	52.8	NA†	(…)	(…)	(…)	(…)
A3	14.0	11.6	28.0	23.8	16.3	15.1	58.6	24.5	(…)	(…)	(…)	(…)
A4	24.9	47.4	97.1	91.4	(…)	(…)	(…)	(…)	(…)	(…)	(…)	(…)
A5	25.0	27.9	51.0	60.3	25.5	39.9	53.4	66.8	(…)	(…)	(…)	(…)
A6	12.2	19.7	27.5	25.0	(…)	(…)	(…)	(…)	(…)	(…)	(…)	(…)
A7	23.1	46.6	85.7	63.0	36.4	56.7	76.9	44.8	37.2	36.1	126.9	50.3
A8	43.1	27.9	41.4	51.5	36.9	23.3	29.4	54.3	26.9	18.8	21.9	39.5
A9	12.6	21.0	35.9	27.6	17.2	27.5	36.1	57.4	16.7	33.0	38.7	49.5
A10	31.4	33.3	31.2	36.0	(…)	(…)	(…)	(…)	22.6	29.0	35.5	34.9
A11	50.1	69.2	108.5	78.3	(…)	(…)	(…)	(…)	(…)	(…)	(…)	(…)
A12	72.5	65.8	91.7	52.7	66.4	59.7	81.4	85.1	54.7	36.3	46.1	57.9
A13	(…)	(…)	(…)	(…)	83.2	67.3	91.0	115.8	(…)	(…)	(…)	(…)
A14	13.4	20.6	23.1	37.8	(…)	(…)	(…)	(…)	(…)	(…)	(…)	(…)
A15	31.3	56.9	30.1	21.3	(…)	(…)	(…)	(…)	(…)	(…)	(…)	(…)
A16	24.4	27.6	50.2	133.5	(…)	(…)	(…)	(…)	44.3	37.0	48.3	29.7
A17	8.7	14.4	30.2	37.1	9.7	11.1	23.8	68.0	8.7	13.3	34.4	17.1

Source: The authors, based on data provided by the Municipal Health Department of Campinas, SP, Brazil.

* (…) = No information, since the hospital does not have intensive care units for the specialty;

† NA: Does not apply, because of the removal of ICU beds to increase the number of beds for adult patients with the coronavirus disease.

In order to compare public and private hospitals, the sample size was the number of months analyzed (n=48). In the public network, median ABHS consumption was 26.9mL/PD (IQR = 17.6) for AICUs, 22.6mL/PD (IQR = 22.3) for PICUs, and 44.0mL/PD (IQR = 30.8) for NICUs. In the private network, median consumption was 46.4mL/PD (IQR = 21.4) for AICUs, 44.5mL/PD (IQR = 25.7) for PICUs, and 33.9mL/PD (IQR = 11.8) for NICUs. The results were statistically significant for AICUs and PICUs in the Mann-Whitney test (p<0.0001). For NICUs, p=0.0210.

**Figure 2:**
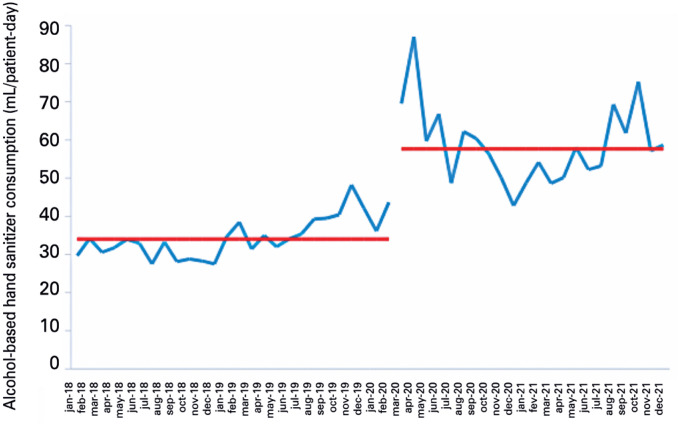
Monthly alcohol-based hand sanitizer consumption in an adult intensive care unit

**Figure 3:**
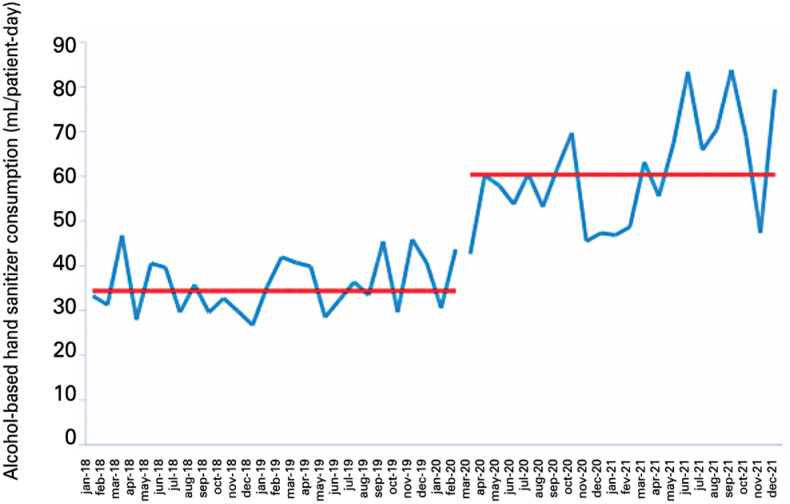
Monthly alcohol-based hand sanitizer consumption in a pediatric intensive care unit

**Figure 4:**
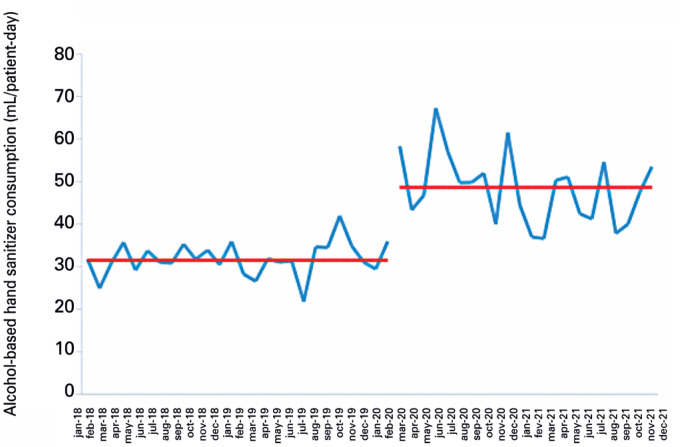
Monthly alcohol-based hand sanitizer consumption in a neonatal intensive care unit

New medians were calculated, with an increase in ABHS consumption of 34.0 to 57.6 mL/PD in AICUs, 34.4 to 60.4 in PICUs, and 31.5 to 48.6 in NICUs (Figures 2 to 4).

## DISCUSSION

Studies have demonstrated that the pandemic contributed to changing the behavior of health professionals in terms of preventive disease transmission measures, thereby increasing adherence to hand hygiene and reducing the incidence of multiresistant bacteria.^(2,26)^ Indeed, the present study revealed a rise in ABHS consumption in ICUs from 24.2 mL/PD in 2018, to 46.6 in 2020.

However, it is important to note the variations in ABHS consumption during the study period. COVID-19 has mainly affected the adult population (Figure 2), whereby AICUs showed a sharp rise in ABHS consumption with the first (between February and November 2020) and second COVID-19 wave in Brazil (between November and April 2021).^(27)^Table 2 shows that some hospitals reported a decline in ABHS consumption in 2021 compared to 2020. Studies carried out during the pandemic using automated hand hygiene monitoring systems have shown that hand hygiene adherence rates increased significantly at the beginning of the pandemic, but gradually decreased back to baseline levels.^(19,20)^

The WHO established an acceptable ABHS consumption for hand hygiene of at least 20mL/PD.^(28)^ Average monthly ABHS consumption in the municipality was above 20mL/PD during the four-year study. However, analysis of each hospital revealed that in 2018, 68.8 and 66.7% of the hospitals obtained results above this index for AICUs and PICUs, respectively, and 81.3 and 88.9%, respectively, in 2019. For NICUs, the percentage was 75.0% in the next two years.

A comparison of national data reported by the National Sanitary Surveillance Agency showed that for AICUs the municipality exceeded most of the Brazilian states.^(29)^ In Brazil, 31.7% of AICUs reached the national ABHS consumption minimum target of 20mL/patient-day in 2018 and 32.6% in 2019. For the remainder, the difference was smaller, 79.2% in 2018 and 76.7% in 2019 for PICUs, and 72.0 and 75.3% in 2018 and 2019, respectively, for NICUs.^(29)^ In 2020 and 2021, all the hospitals in the municipality exceeded the minimum target, with a subsequent rise in mean and median ABHS consumption in the ICUs, as well as in other studies.^(2,30)^

In general, patients in intensive care require greater care and workload, which consequently increase the indications and opportunities for hand hygiene. Given the effectiveness of ABHS in reducing the microbial load of hands, with volumes varying between 1.75 and 3.5mL per application,^(22)^ that is, for each indication involving the 5 moments of hand hygiene, it was shown that hospitals leave much to be desired in safe direct intensive care. A study conducted in Germany involving 1068 hospitals reported mean consumption of 137.4mL/PD in 2018 for AICUs, 169 for PICUs and 176 for NICUs,^(31)^ that is, a 4 to 5-fold higher consumption than that of Campinas in the same year. According to the literature, the fact that newborns have an immature immunological system, that is, they are more susceptible to serious complications, may contribute to greater concern on the part of health professionals and stimulate hand hygiene.^(31)^ As such, it is believed that the minimum target of 20mL/PD could be reassessed for critical units.

Alcohol-based hand sanitizer consumption differed between hospitals, where the AICUs and PICUs of private hospitals used more than their public counterparts. Studies carried out at Brazilian hospitals demonstrated that despite meeting basic infrastructure indicators, hand hygiene adherence was low at the institutions assessed, but better at private hospitals.^(32)^

In addition to the physical structure of the units, hand hygiene adherence was also influenced by the use of gloves, work regime, and patient safety.^(33)^ The use of gloves was considered a risk factor for nonadherence to the 5 moments of hand hygiene, especially before aseptic procedures and after exposure to body fluids. Safety was directly proportional to hand hygiene, given that temporary professionals were more perceptive than those hired based on their civil service examination results.^(33)^

Use of gloves was also associated with low hand hygiene adherence in 47% of missed opportunities.^(34)^ The authors used direct observation and found that even during the pandemic, adherence was very low, with only 6.1% (confidence interval= 3.4-8.7) performed using the correct technique, within the minimum recommended time.^(34)^ Analysis of the infrastructure revealed that the unit had an insufficient number of ABHS dispensers at the point of care, which was difficult to access because the dispensers were located behind patients’ beds or equipment, as well as problems in supplying the product and dispenser malfunctioning.^(34)^

According to the Society for Healthcare Epidemiology of America guidelines, strategies to obtain a satisfactory adherence level include the following: accessible and functional alcohol dispensers, available sink, paper towels, training and programs, qualified observers to monitor professionals, and data analysis.^(10)^ During this period, the different infrastructure between public and private hospitals should be considered, since any deficit in basic infrastructure and supplies can have a negative impact on adherence to hand hygiene. An investigation conducted by the WHO on hand hygiene involving 88 countries in 2019, revealed a 1:3 ratio of health units that provided hand hygiene materials to the patients.^(35)^ The high demand for supplies at peak hospitalization rates during the pandemic may have contributed to the lack of resources in public institutions.^(17)^

This study exhibited limitations related to shortcomings inherent to the indirect monitoring of ABHS consumption, precluding analysis of the hand hygiene technique, or whether it was executed in timely fashion during patient care, which can hamper assertive feedback or targeted improvements. In addition, this study used the indirect documentation technique, the researchers did not have access to the hospitals, and the spreadsheet used as data source did not contain information on how each hospital quantifies the amount of ABHS consumed.

It is important to note that this is a pioneering study on the interim assessment of a Brazilian municipality, comparing ABHS consumption before and during the COVID-19 pandemic. There was a gradual improvement between 2018 and 2020 at the ICUs assessed. In 2021, the ABHS consumption indicator were sustained for AICUs and PICUs, which could be interpreted with caution, since analysis of hospitals individually makes it possible to identify the shortcomings that will lead to specific local strategies.

It is important to underscore that preventing and controlling infection and antimicrobial resistance is one of the strategies to eliminate avoidable harm during health care, improving patient safety and healthcare quality, as part of the strategic goals of the 2021-2030 Global Patient Safety Action Plan.^(36)^

Public policies and clinical practices for infection prevention and control cover all areas of the healthcare system and are at the heart of healthcare safety and quality, global health security and the response to health emergencies.^(3,5)^

Hand hygiene is the primary infection prevention, antimicrobial resistance and emerging health threat component. As such, the combination of more than one auditing method may produce a more accurate result, reduce the risk of bias and provide more concrete data to devise effective strategies.

The results of the present study can be used to guide surveillance measures and establish intervention strategies that can assess the hand hygiene behavior of health professionals, aimed at improving the healthcare results as a whole. Future studies are needed to determine whether the alcohol-based hand sanitizer consumption indicator obtained in the municipality will be sustained with the end of the pandemic.

## CONCLUSION

It can be concluded that the COVID-19 pandemic increased alcohol-based hand sanitizer consumption in the adult, pediatric and neonatal intensive care units, compared to the same pre-pandemic period. This increase was significant between the adult intensive care units and pediatric intensive care units of private hospitals, when compared to their public counterparts.
